# Clipping versus coiling in posterior circulation intracranial aneurysms: a meta-analysis

**DOI:** 10.1186/s41016-019-0163-x

**Published:** 2019-06-19

**Authors:** Eleni Tsianaka, Abdullah Al-Shawish, Alexander Potapov, Kostas Fountas, Michael Spyrou, Nikolay Konovalov

**Affiliations:** 1 Department of Neurosurgery, University Hospital of Larissa, Viopolis, 41111 Larissa, Greece; 2 Department of Neurosurgery, Ibn Sina Hospital, Sabah Medical Area, Shuwaikh, Kuwait; 3 Department of Neurotraumatology, “N.N. Burdenko” Neurosurgery Institute, 16 4th Tverskaya – Yamskaya Str., Moscow, Russia; 4 Department of Neurosurgery, Ygeia Private Hospital, Golgon 33, 3025 Limassol, Cyprus; 5 Department of Spine Neurosurgery, “N.N. Burdenko” Neurosurgery Institute, 16 4th Tverskaya – Yamskaya Str., Moscow, Russia

**Keywords:** Clipping, Coiling, Intracranial aneurysms, Meta-analysis, Posterior circulation

## Abstract

**Background:**

Posterior circulation intracranial aneurysm (IA) treatment remains challenging, due to the anatomy of the area and the high rupture possibility. Endovascular treatment seems to be more suitable for these aneurysms, but studies focused on endovascular treatment demonstrate a high rate of re-intervention needing. A meta-analysis might offer a clearer view, being useful in a more effective treatment planning.

**Methods:**

A systematic search was performed, using the PubMed database platform. The final article pool contained 20 articles. Studied parameters were operative mortality, late mortality, permanent neurologic deficit (PND), and the need for re-intervention (Re-int). We divided patients into two subgroups, those with ruptured and those with unruptured aneurysm. Statistical analysis was performed using appropriate software.

**Results:**

In the total population (645 patients), there was a superiority of coiling over clipping in terms of PND and of coiling in terms of Re-int. As regards mortality, there was no clear superiority of one method over the other.

**Conclusions:**

The current study came to the conclusion that there is a superiority of coiling over clipping in terms of PND. On the other hand, clipping seems to be superior to coiling in terms of the need for re-intervention. As regards mortality (both operative and late), there is no clear superiority of one method over the other. Studying subgroups of patients (ruptured and unruptured posterior circulation IAs), in terms of PND, there is no superiority of one method over the other. The same goes for Op-Mo on ruptured aneurysms.

## Background

Intracranial aneurysms (IAs) are not such a rare pathological condition, as they affect 5–10% of the general population [1], while they are responsible for about 80% of non-traumatic subarachnoid hemorrhages (SAH) [2]. 3.8–15% of all IAs are related to posterior circulation (vertebral artery (VA), basilar artery (BA), posterior inferior cerebellar artery (PICA), anterior inferior cerebellar artery (AICA), superior cerebellar artery (SCA), and posterior cerebral artery (PCA)) [3]. Taking into account all these facts, posterior circulation IAs affect about 0.19–1.5% of the general population. Most of the posterior circulation aneurysms appear to be located at the basilar apex (10% of all IAs) [4]. Their treatment remains challenging, due to the anatomy of the area (brainstem and cranial nerves) and the higher rupture possibility, compared with anterior circulation IAs [5]. It is no surprise that posterior circulation surgical treatment appears to have higher morbidity and mortality rate, compared to anterior circulation aneurysms [6]. In this way, the endovascular treatment seems to be more suitable for these aneurysms. But, is that really the key to the successful treatment of posterior circulation IAs? Studies focused on endovascular treatment of the IAs demonstrate a higher rate of re-intervention needing for posterior circulation aneurysms, in contrast with those of anterior circulation [7]. Successful treatment concept includes many factors, not only during the surgical or endovascular procedure, but at the post-operative time too, in terms of mortality, morbidity, and re-intervention needing. In order for these issues to become clearer, there were some studies conducted worldwide. Some of these focus on the efficacy of either surgical or endovascular treatment, whereas some others on comparing these two treatment strategies. Generally, the cohort in those studies was small, due to the low incidence of this kind of aneurysms in the general population. After taking into account the inclusion and exclusion criteria, the cohort remained even smaller. A meta-analysis might offer a clearer view of those issues, being useful in a more effective treatment planning.

## Material and methods

The strategic plan for searching the appropriate material was based on the Preferred Reporting Items for Systematic Reviews and Meta-Analyses (PRISMA) [8]. A systematic search was performed using the PubMed database platform (last search on May 23, 2017). The keywords which were used on the searching process were as follows: intracranial aneurysms, posterior circulation, clipping, and coiling. In addition, various filters were used, in order for our searching to be more focused. So, we limited our search to classical articles, clinical studies, clinical trials, controlled clinical trials, and multicenter studies. The text format included only full-text available articles in English language. In order to avoid major variabilities regarding the operative techniques, we decided to apply a time filter excluding articles older than 1997. All articles were referred to human adults. There were 85 articles that were identified at that stage. A wider manual electronic search, based on reference lists from other database platforms, such as MEDLINE, was performed, in order for the list of the included articles to be as complete as possible. The final target was to form an article pool, which would refer to the results of the clipping posterior circulation IAs, compared to the coiling of them. Towards the end of our search, we performed duplicate checking. Finally, there were 132 candidate articles selected.

**Fig. 1:**
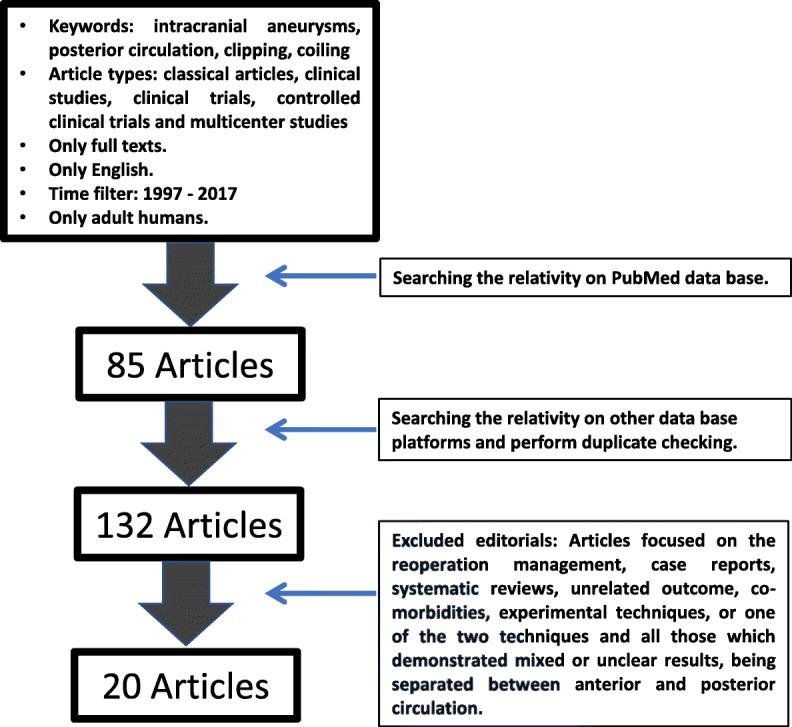
Article selection algorithm

Our next step was the title and abstract screening of those 132 articles. Two of the authors performed screening by working independently from each other. A third one was acting as a referee, whenever there was a conflict between the above reviewers. The inclusion criteria for the articles in order to be characterized as appropriate were (a) to compare the outcomes between two techniques, (b) to demonstrate at least one of the studied outcomes, (c) to refer to classical coiling treatment, without additional or modified devices (such as stent-assisted coiling), in order for our cohort to be more homogenized. The articles which were excluded from that article pool were those which focused on the reoperation management, case reports, systematic reviews, unrelated outcome, co-morbidities, experimental techniques, or one of the two techniques and all those which demonstrated mixed or unclear results, being separated between anterior and posterior circulation. The final article pool contained 20 articles, as appropriate for our meta-analysis [7–26] (Fig. 1).

Our next step was data extraction from that final article pool. In that procedure, 3 of the co-authors were involved. They worked independently on that issue. All related data (first author, country, year of publication, study covered period, cohort size, operative mortality, permanent neurologic deficit, late mortality, and need for re-intervention) from these articles were included in a standard form. As regards mortality during the first 30 days, it was defined as operative mortality (Op-Mo) and after that as late mortality (La-Mo). The permanent neurologic deficit (PND) includes every focal or global neurologic deficit, presenting permanently after discharge. The re-intervention (Re-int) could be on a previous coiled or clipped aneurysm and could be either of the same kind or not. The follow-up was based on, at least, a 6 month-period after SAH or intervention. All disagreements on results were resolved in a common meeting of the 3 co-authors who took part in the data extraction procedure (Table 1).

**Table 1:** Posterior circulation IA data extraction

Posterior circulation IA data extraction														
First author	Country	Publication year	Covered period (years)	Total patients	Patients clipping	Patients coiling	Op-Mo clipping	Op-Mo coiling	PND clipping	PND coiling	La-Mo clipping	La-Mo coiling	Re-int clipping	Re-int coiling
Seifert V	Germany	2001	7	24	23	1	2	0	10	0	0	0	–	–
Lusseveld E	Netherlands	2002	16	88	44	44	5	2	8	3	–	–	–	–
Uhl E	Germany	2003	7	3	1	2	0	0	0	2	0	0	0	1
Sheah K	Singapore	2005	14	6	1	5	0	0	0	0	0	0	0	1
Sandalcioglu I	Germany	2005	5	22	9	13	0	1	3	5	1	1	–	–
Molyneux A	The UK	2005	3	58	34	24	–	–	–	–	15	4	–	–
Kocaeli H	The USA	2009	7	12	6	6	0	1	2	2	0	0	0	1
Sung KS	Korea	2009	4	34	21	13	0	0	4	1	0	0	0	2
Jin SC	Korea	2009	8	77	34	43	1	6	6	4	0	0	0	9
Kaku Y	Japan	2010	3	11	4	7	0	1	–	–	–	–	–	–
Nourbakhsh A	The USA	2010	10	14	11	3	–	–	8	2	1	0	2	0
Zhou Y	Japan	2010	5	4	2	2	0	1	1	0	–	–	–	–
Tokimura H	Japan	2011	10	23	19	4	0	0	2	0	–	–	–	–
Hong YH	Korea	2011	15	20	11	9	3	3	5	3	0	0	–	–
Spetzler R	The USA	2013	4	69	40	29	0	0	–	–	0	1	–	–
Sharma M	The USA	2013	5	20	5	15	0	0	1	0	0	0	–	–
Bacigaluppi S	Italy	2013	10	18	1	17	0	2	1	5	0	0	0	3
Sharma S	India	2014	9	45	19	26	3	6	–	–	0	0	0	1
Jang EW	Korea	2015	14	82	17	65	0	1	1	2	–	–	–	–
Sejkorova A	Czech Republic	2015	9	15	9	6	2	2	1	1	0	0	1	2
Total patients				645	311	334	16	26	53	30	17	6	3	20

*–* data not reported

Furthermore, we divided patients into two subgroups, those with ruptured and those with unruptured aneurysm of the posterior circulation, and we formed a table, following the same procedure as above. The articles with unclear aneurysm state (ruptured or unruptured) were excluded from that procedure (Table 2).

**Table 2:** Subgroups: unruptured and ruptured IA data extraction

First author	Country	Publication year	Covered period (years)	Total patients	Patients clipping	Patients coiling	Op-Mo clipping	Op-Mo coiling	PND clipping	PND coiling	La-Mo clipping	La-Mo coiling	Re-int clipping	Re-int coiling
Unruptured posterior circulation IA data extraction														
Kocaeli H	The USA	2009	7	7	3	4	0	0	1	2	0	0	0	0
Sung KS	Korea	2009	4	7	1	6	0	0	0	0	0	0	–	–
Jin SC	Korea	2009	8	28	17	11	1	0	3	1	0	0	–	–
Tokimura H	Japan	2011	10	2	1	1	0	0	0	0	–	–	–	–
Sharma M	The USA	2013	5	20	5	15	0	0	1	0	0	0	–	–
Bacigaluppi S	Italy	2013	10	2	1	1	0	0	1	0	0	0	0	0
Jang EW	Korea	2015	14	82	17	65	0	1	1	2	–	–	–	–
Total patients				148	45	103	1	1	7	5	0	0	0	0
Ruptured posterior circulation IA data extraction														
Seifert V	Germany	2001	7	22	21	1	2	0	9	0	0	0	–	–
Lusseveld E	Netherlands	2002	16	88	44	44	5	2	8	3	–	–	–	–
Uhl E	Germany	2003	7	3	1	2	0	0	0	2	0	0	0	1
Sheah K	Singapore	2005	14	6	1	5	0	0	0	0	0	0	0	1
Sandalcioglu I	Germany	2005	5	21	8	13	0	1	3	5	1	3	–	–
Kocaeli H	The USA	2009	7	5	3	2	0	1	1	0	0	0	0	1
Sung KS	Korea	2009	4	27	20	7	0	0	–	–	–	–	–	–
Jin SC	Korea	2009	8	49	17	32	0	6	3	3	0	0	–	–
Kaku Y	Japan	2010	3	11	4	7	0	1	–	–	–	–	–	–
Nourbakhsh A	The USA	2010	10	13	10	3	–	–	8	2	0	0	2	0
Zhou Y	Japan	2010	5	4	2	2	0	1	1	0	–	–	–	–
Tokimura H	Japan	2011	10	21	18	3	0	0	2	0	–	–	–	–
Hong YH	Korea	2011	15	20	11	9	3	3	5	3	0	0	–	–
Spetzler R	The USA	2013	4	69	40	29	0	0	–	–	0	1	–	–
Sejkorova A	Czech Republic	2015	9	4	1	3	1	2	0	1	0	0	0	1
Total patients				363	201	162	11	17	40	19	1	4	2	4

*–* data not reported

In order for the risk of bias of our article pool to be determined, a quality assessment tool was used (Newcastle Ottawa Scale (NOS)) [29, 31]. Two of the co-authors conducted the quality control, and the final solutions were given at a meeting of four of the authors (Table 3).

**Table 3:** NOS quality assessment of final article pool

Study	Selection			Outcome not present at the start of the study	Comparability based on the design or analysis	Outcome		Adequacy of follow-up	Score
	Representativeness	Selection	Ascertainment of exposure			Assessment of outcome	Follow-up long enough		
Seifert et al.	●	●	●	●	●	●	●	●	8
Lusseveld et al.	●	●	●	●	●●	●	–	●	8
Uhl et al.	●	●	●	●	●	●	●	●	8
Sheah et al.	●	●	●	●	●	●	●	●	8
Sandalcioglu et al.	●	●	●	●	●	●	●	●	8
Molyneux et al.	●	●	●	●	●	●	●	●	8
Kocaeli et al.	●	●	●	●	●	●	●	●	8
Sung et al.	●	●	●	●	●●	●	●	●	9
Jin et al.	●	●	●	●	●●	●	●	●	9
Kaku et al.	●	●	●	●	●	●	–	●	7
Nourbakhsh et al.	●	●	●	●	●	●	●	●	8
Zhou et al.	●	●	●	●	●	●	–	●	7
Tokimura et al.	●	●	●	●	●	●	–	●	7
Hong et al.	●	●	●	●	●	●	●	●	8
Spetzler et al.	●	●	●	●	●●	●	●	●	9
Sharma M et al.	●	●	●	●	●●	●	●	●	9
Bacigaluppi et al.	●	●	●	●	●●	●	●	●	9
Sharma S et al.	●	●	●	●	●●	●	●	●	9
Jang eta al.	●	●	●	●	●●	●	–	●	8
Sejkorova et al.	●	●	●	●	●●	●	●	●	9

NOS scale awards each item with one point (•). Comparability is awarded with a maximum of two points (••). In case an article doesn’t meet a criterion, it gains no points (-). A total score less than 5 for an article means high risk of bias

The statistical analysis was performed using the appropriate software (RevMan 5, The Nordic Cochrane Centre, The Cochrane Collaboration, Copenhagen, Denmark). The dichotomous outcomes were based on a meta-analysis, using the calculation of the odds ratio (OR), with 95% interval of confidence (CI). OR is defined as the odds of an event occurring in the clipping group, divided by the odds of the same event occurring in the coiling group. OR values < 1 support open surgical repair by clipping of the aneurysm. Statistical significance is identified when *p* < 0.05, provided that value 1.0 is not included in 95% CI. Chi-square test was used for the determination of heterogeneity (*p* < 0.10 or *I*^2^ > 50% support heterogeneity) [30].

## Results

After the initial searching, 132 articles were found to be candidates for further analysis. After applying all exclusion and inclusion criteria, there were 20 articles left into the final article pool [8–29]. Those articles were screened in detail (Table 1). The total number of patients included in those 20 articles was 645 (311 in the clipping group and 334 in coiling). Eighteen articles gave information about Op-Mo, but only 12 of them demonstrated events [2–26]. The data about PND were included in 16 articles, 15 of which demonstrated events [11, 12, 14, 15, 17, 19, 21, 23, 26–28]. The next parameter was La-Mo, for which there was found information in 15 articles, but some events are demonstrated in only 4 articles [10, 18, 22, 23]. The last parameter was performing of re-intervention, for which we had reports from nine articles, all of which included events [12–15, 20, 23–26, 29] (Table 1). As about the posterior circulation unruptured IA subgroup, there were 7 articles giving information. There were 148 total patients included (45 clipping and 103 coiling) [14, 15, 17, 19, 21, 25, 29] (Table 2). All of them contain information about Op-Mo and PND but only two of them demonstrate events about Op-Mo [18, 25] and 5 about PND [14, 19, 21, 25, 29]. About La-Mo and Re-int, there are no events mentioned. The second subgroup was that of posterior circulation ruptured IAs. In that category, there were 15 articles giving related information [11–18, 23, 24, 26–29]. There is information about Op-Mo in 14 of them, but only 9 of them contain events [11, 14, 16, 22, 24, 26–29]. Twelve contain information about PND and 11 of them demonstrate events [11, 12, 14, 17, 22–24, 26–29]. Out of the 10 articles giving information about La-Mo, there is only two including an event [18, 22]. Finally, 5 articles talk about Re-int and all of them demonstrate events [12–14, 23, 26] (Table 2).

**Fig. 2:**
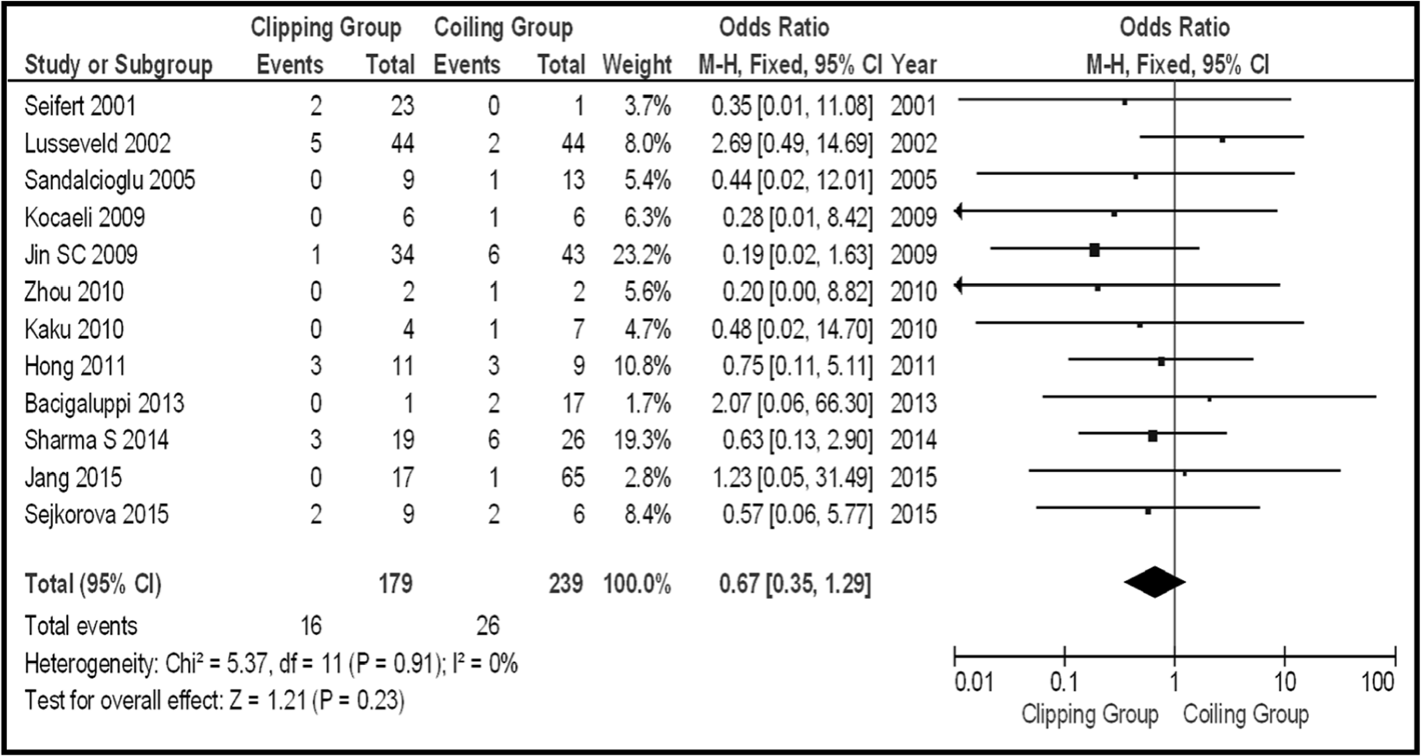
OR forest plot, showing Op-Mo in clipping versus coiling, on posterior circulation IAs (unruptured and ruptured)

**Fig. 3:**
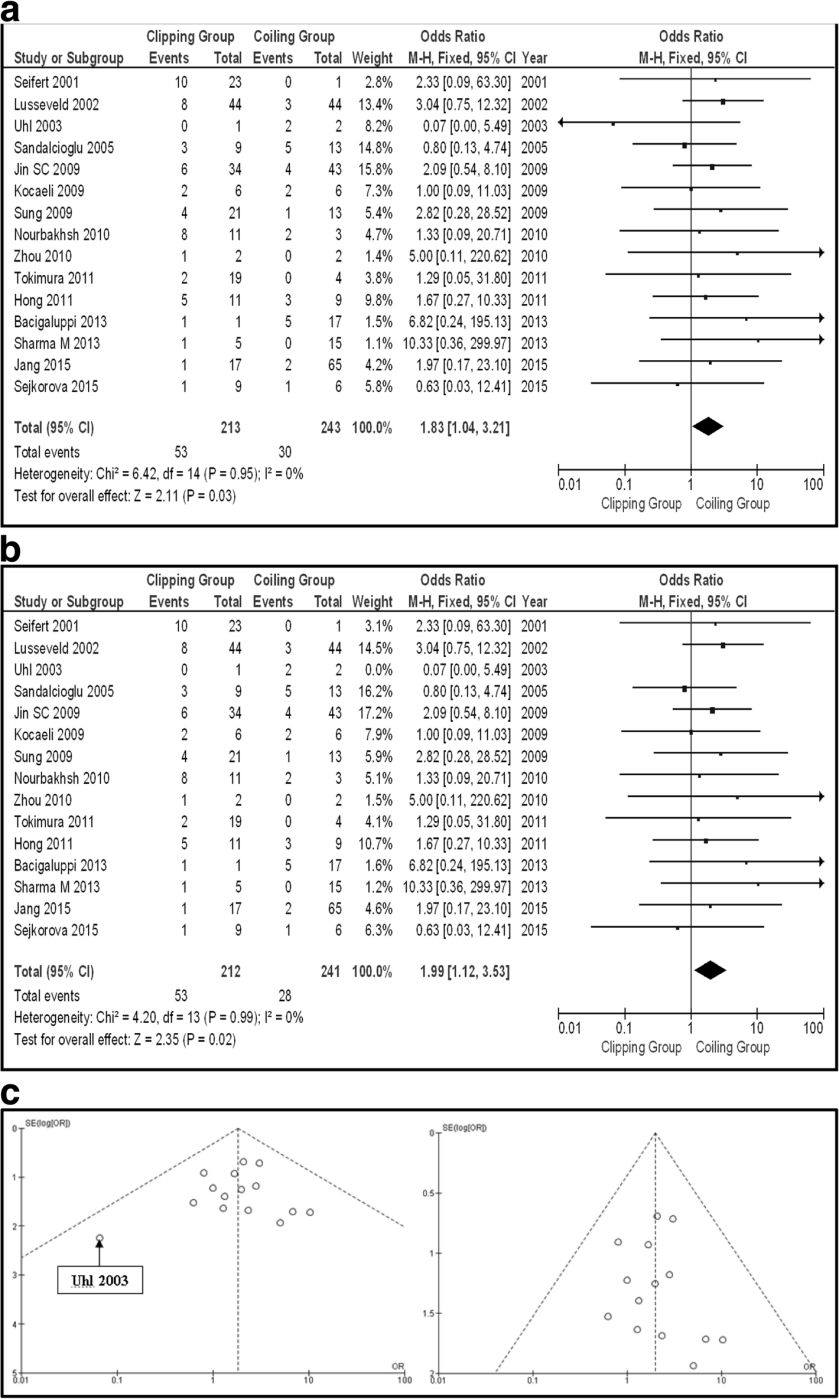
**a** OR forest plot, showing PND in clipping versus coiling, on posterior circulation IAs (unruptured and ruptured). **b** OR forest plot, showing PND in clipping versus coiling, on posterior circulation IAs (unruptured and ruptured), excluding “Uhl 2003” study. **c** Funnel plots of the PND in the total group of patients (unruptured and ruptured aneurysms), with (left) and without (right) “Uhl 2003” article

**Fig. 4:**
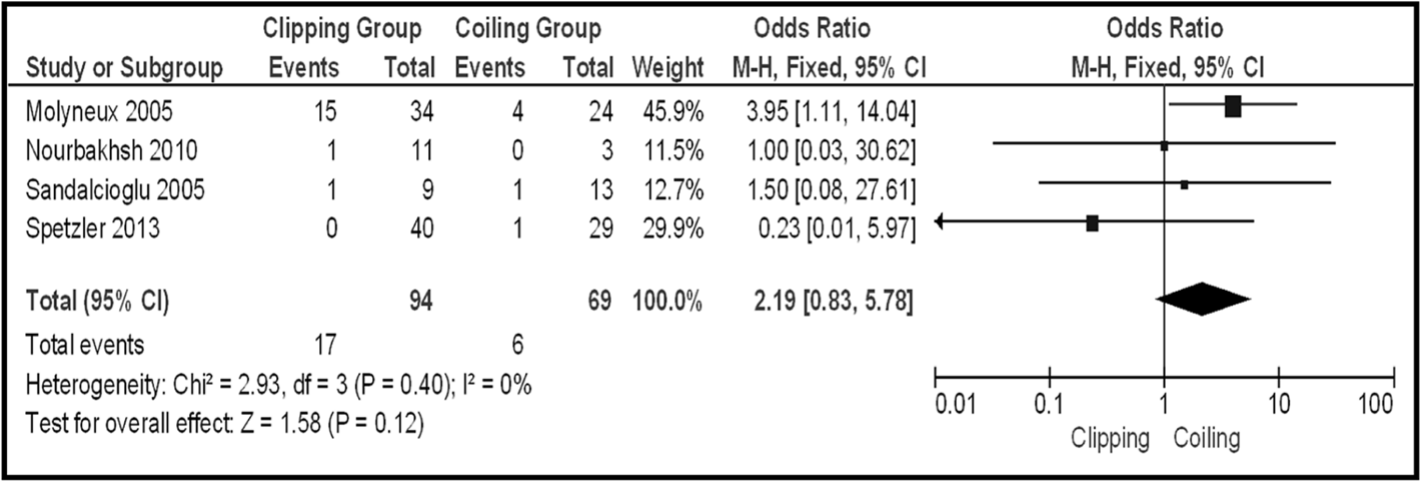
OR forest plot, showing La-Mo in clipping versus coiling, on posterior circulation IAs (unruptured and ruptured)

**Fig. 5:**
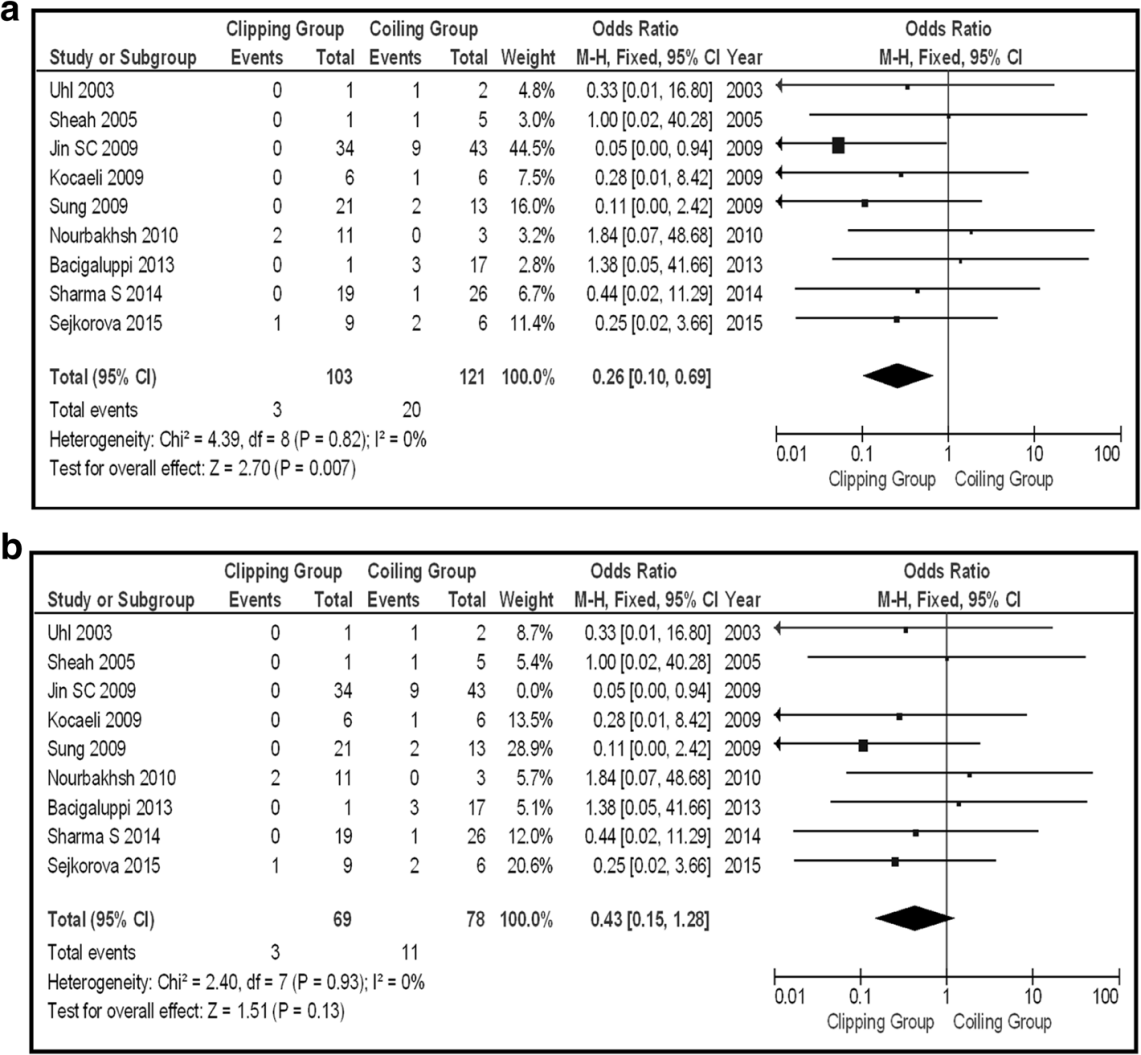
**a** OR forest plot, showing Re-int in clipping versus coiling, on posterior circulation IAs (unruptured and ruptured). **b** OR forest plot, showing Re-int in clipping versus coiling, on posterior circulation IAs (unruptured and ruptured), excluding “Jin 2009” study

In the total group of patients (unruptured and ruptured), 12 series provided information about Op-Mo (total 418 patients, 179 in the clipping group, and 239 in the coiling group). Sixteen deaths were in the clipping group and 26 in the coiling group. The final results saw no potential significant difference between the two groups (OR 0.67, CI 95% 0.35–1.29, and *p* = 0.23), with no heterogeneity (*p* = 0.91 and *I*^2^ = 0%) (Fig. 2). In the same group, the incidence of PND was provided by 15 articles (456 patients in total, 53 clipping, and 30 coiling). There were 53 PND events in the clipping group and 30 in the coiling one. The pooled results had to demonstrate the statistically significant difference between surgical repair by clipping and coiling (OR 1.83, CI 95% 1.04–3.21, and *p* = 0.03) with no heterogeneity (*p* = 0.95 and *I*^2^ = 0%). Testing the sensitivity, we were removing one study at a time. After removing the “Uhl 2003” [12], there was additional statistical significant superiority of coiling group, over clipping (OR 1.99, CI 95% 1.12–3.53, and *p* = 0.02), without heterogeneity (*p* = 0.99 and *I*^2^ = 0%) (Fig. 3a, b). Looking at the funnel plot of the same parameter, we found that the study results without “Uhl 2003” article displayed better dispersion, with very low publication bias, in contrast with the same study including “Uhl 2003” article (Fig. 3c). Information regarding La-Mo was available only from 4 articles (total 163 patients, 94 clipping, and 69 coiling)—17 events on the clipping group and 6 on the coiling one—showing no statistically significant superiority of one over the other method (OR 2.19, CI 95% 0.83–5.78, and *p* = 0.12) (Fig. 4). Sequential removal of studies one by one showed statistically significant superiority of coiling over clipping, by excluding “Spetzler 2013” [18]. Re-int was reported by 9 articles, totaling 224 patients (103 clipping and 121 coiling). The vast majority of Re-int took part in the coiling group (20 patients). From the clipping group, there were only 3 patients needing re-intervention. The results of the analysis showed a statistically significant difference between the two groups (OR 0.26, CI 95% 0.10–0.69, and *p* = 0.007), without heterogeneity (*p* = 0.82 and *I*^2^ = 0%), emphasizing clipping superiority (Fig. 5a). Sensitivity testing, by sequential exclusion of the studies one at a time, showed that there is no statistical significance of the clipping superiority, by excluding “Jin 2009” [29] (Fig. 5b). That was expectable, because “Jin 2009” series represents 44.5% of the results (Fig. 5a).

**Fig. 6:**
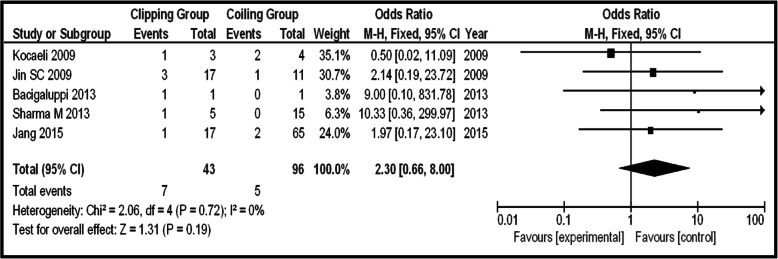
OR forest plot, showing PND in clipping versus coiling, on posterior circulation unruptured IAs

As regards the subgroup of patients with unruptured posterior circulation IAs, Op-Mo events are demonstrated in only two articles [21, 29]. In that case, there can be no statistical analysis about that factor. The same goes for La-Mo and Re-int, because there are no series which demonstrate events on these parameters. The only factor which can be studied is PND. There were 5 articles providing information on PND events (total 139 patients, 43 clipping, and 96 coiling). Seven PND events occurred on the clipping group and 5 on the coiling one. The statistical analysis showed no potential significant difference between the two groups (OR 2.30, CI 95% 0.66–8.00, and *p* = 0.19), providing no heterogeneity (*p* = 0.72 and *I*^2^ = 0%) (Fig. 6).

**Fig. 7:**
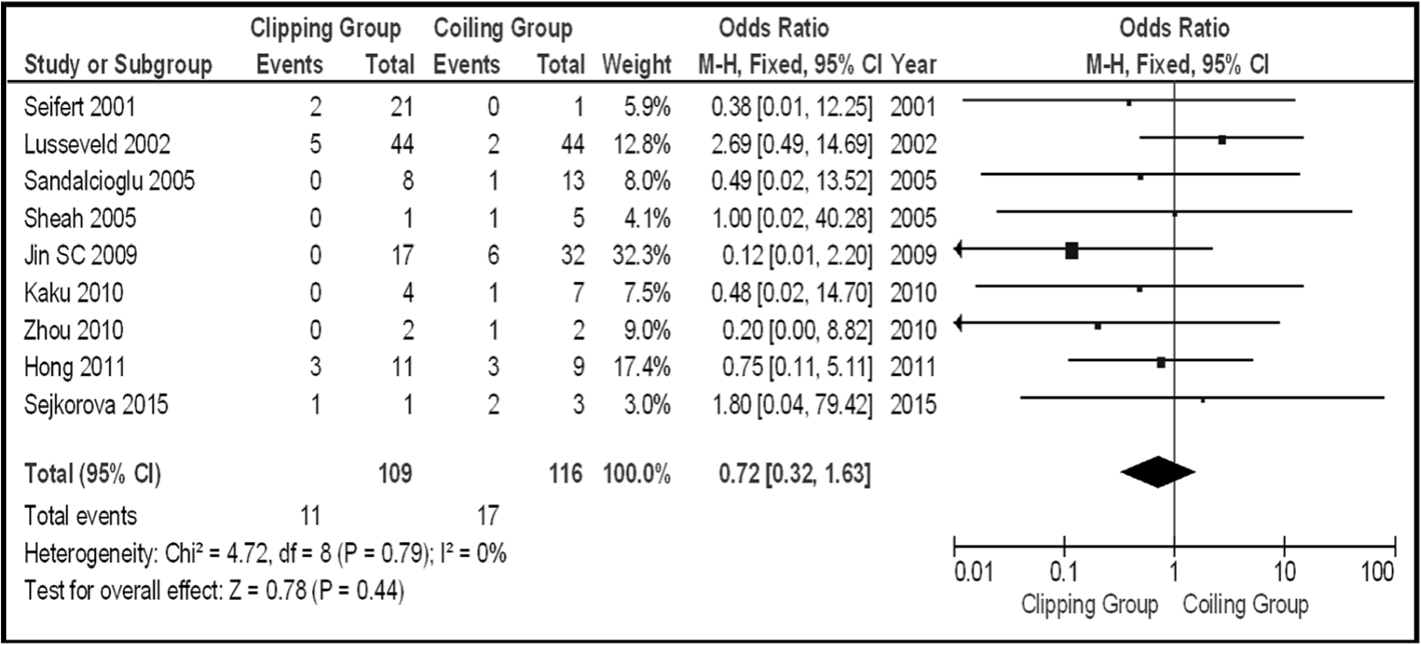
OR forest plot, showing Op-Mo in clipping versus coiling, on posterior circulation ruptured IAs

**Fig. 8:**
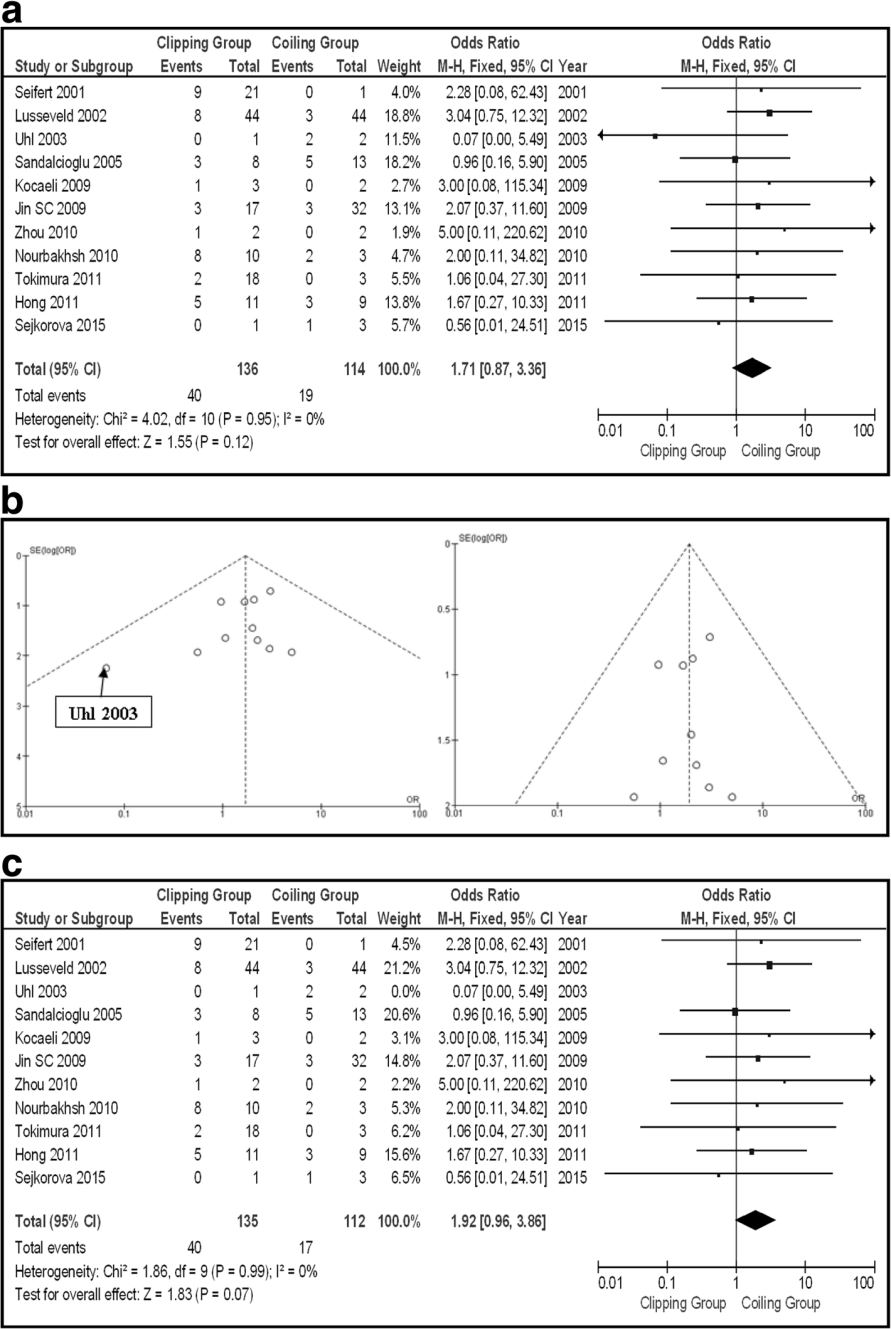
**a** OR forest plot, showing PND in clipping versus coiling, on posterior circulation ruptured IAs. **b** Funnel plots of the PND in the group of patients with ruptured aneurysms, with (left) and without (right) “Uhl 2003” article. **c** OR forest plot, showing PND in clipping versus coiling, on posterior circulation ruptured IAs, without “Uhl 2003” series

The second subgroup was that of the ruptured posterior circulation IAs. In that group of patients, any information about Op-Mo occurred events was given in 9 articles (total 225 patients, 109 clipping, and 116 coiling). There were 11 occurred events in the clipping group and 17 in coiling one. There was no statistically significant difference between the two groups (OR 0.72, CI 95% 0.32–1.63, and *p* = 0.44), showing no heterogeneity (*p* = 0.79 and *I*^2^ = 0%) (Fig. 7). Eleven series gave information about occurred PND events (total 250 patients, 136 clipping, and 114 coiling). There were 40 PND events in the clipping group and 19 in the coiling one, showing no statistically significant difference between the two groups (OR 1.71, CI 95% 0.87–3.36, and *p* = 0.12), supporting no heterogeneity (*p* = 0.95 and *I*^2^ = 0%) (Fig. 8a). Studying the funnel plot for the same parameter, we found that the study results which excluded “Uhl 2003” series displayed better dispersion, with very low publication bias, in contrast with the same study including the “Uhl 2003” article (Fig. 8b). That change does not affect the statistical significance of the results of the PND parameter (Fig. 8c). La-Mo occurred events are demonstrated only in two studies (total 90 patients, 48 clipping, and 42 coiling), including 1 event on the clipping group and 4 on the coiling one, so there is no opportunity for further analysis [18, 22]. As about Re-int, there were five articles giving information about occurred events, but the population is very small (31 patients in total, 16 clipping, and 15 coiling), so further analysis is unavailable. We can only note that there were 2 Re-int events on the clipping and 4 on the coiling group.

Sensitivity was tested using the leave-one-out method. Except for the PND and Re-int factor in total patient group (unruptured and ruptured aneurysms), as it has previously been described, there was no other effect of this action. Additionally, we performed publication bias potential expectancy, using funnel plots. Two cases which required analysis were PND in total and in ruptured aneurysm group of patients, as mentioned above.

## Discussion

Posterior circulation aneurysms demonstrate a higher risk of bleeding and they appear to have a larger dome size, in contrast with aneurysms of anterior circulation [32]. Considering the anatomical position of the posterior circulation IAs, surgical management is very challenging. On the other hand, endovascular repair of these aneurysms seems to be a kind of solution, using minimally invasive techniques. There are many studies demonstrating one of these two techniques, in terms of effectiveness and safety. Some of these try to compare surgical (clipping) and endovascular (coiling), but the cohort is small and the evidence of superiority is low. Studies are small, primarily, due to the relative rarity of these aneurysms (3.8–15% of all IAs) [3]. Our study is a meta-analysis, combining all existing comparative studies between the two techniques, under specified criteria, in order to find out the possible superiority of the one method over the other, on posterior circulation IAs. By searching the database, there was no other meta-analysis, using the criteria we used. The International Subarachnoid Aneurysm Trial (ISAT) was a study with great expectations, but unfortunately, it provides lack of information about mortality and morbidity of posterior circulation IAs, mainly due to the subgroup’s studying in different directions. It is characteristic that only 2.7% of the total patients in that study belong to the posterior circulation subgroup. So, that study did not meet our inclusion criteria [9, 10]. International Study of Unruptured Intracranial Aneurysms (ISUIA) [33] is a large and well-organized multi-centered clinical study, dealing with unruptured aneurysms, but the endovascular group included different kind of endovascular procedures, not only simple coiling. Because of that, ISUIA was excluded from our study.

Regarding the total group of patients (both ruptured and unruptured), in terms of Op-Mo and La-Mo, there was no superiority of the one method over the other (Figs. 2 and 4). It is not the first time, that is surprisingly concluded no superiority of the one method over the other in terms of mortality, studying posterior circulation IAs [34]. In unruptured aneurysm subgroup, it was not possible for these two outcome parameters to be studied, because of lack of data. In the ruptured aneurysm subgroup, the results about Op-Mo were not statistically significant (Fig. 7), and about La-Mo, not available for analysis, because of lack of data.

As about PND, coiling was superior to clipping in the total group, but the same does not go for the subgroups too. Both ISAT and ISUIA showed endovascular techniques superiority in terms of morbidity and neurological outcome for posterior circulation IAs [9, 10, 33]. Under these circumstances, the elderly population, suffering serious comorbidities, and those with poor neurological status seem to be of greater benefit under coiling treatment. A larger sample could clarify the situation on subgroups (marginal not statistically significant comparison on the group of ruptured aneurysms) (Figs. 3a, b, 6, and 8a, b).

On the other hand, clipping seems to be superior to coiling in terms of Re-int, in the total group of patients (Fig. 5a). ISAT long-term results provide us with the information that re-bleeding rate is much higher in patients with recurrent aneurysms (regarding incomplete coiling) than in patients with definitive occlusion of their aneurysms. Additionally, the mortality rate after re-bleeding is highly increased (70%) [35]. Generally, younger patients seem to benefit more from using microsurgical techniques, because better long-term results are achieved, considering the need for re-intervention. The superiority of microsurgical treatment for younger patients is well described by other studies too, especially when the aneurysm is small (< 9 mm) [36, 37]. Applying the leave-one-out method to our study suggests low robust of the results for Re-int outcome factor (Fig. 5b). The subgroup study is a method which can eliminate low robust factor, but in both subgroups (unruptured and ruptured aneurysms), there was a lack of data providing events and the comparative populations were small.

A previous meta-analysis, comparing these two techniques on anterior circulation IAs, showed no absolute superiority of one technique over the other, stating that it is better to act on a case by case management direction [38]. In the management selection of the posterior circulation IAs, there are many factors which must be taken under consideration, like the clinical status of the patient, anatomy of the aneurysm, doctor’s experience, technical availability, comorbidities, age of the patient, and previous bleeding. In the future, a larger sample study for comparison, taking into consideration different, more complex endovascular techniques, different surgical procedures and specific particularities on posterior circulation aneurysms anatomy can give even more concrete results, even at the subgroup level.

## Conclusion

The current study came to the conclusion that there is a superiority of coiling over clipping in terms of PND. On the other hand, clipping seems to be superior to coiling in terms of the need for re-intervention. As about mortality (both operative and late), there is no clear superiority of the one method over the other. Studying subgroups of patients (ruptured and unruptured posterior circulation IAs), in terms of PND, there is no superiority of the one method over the other. The same goes for Op-Mo on ruptured aneurysms. The subgroup study needs larger comparative cohorts, in order to come in stronger generalized conclusions, as can be seen on Table 4 (conclusion on the comparison between coiling and clipping, on the management of posterior circulation IAs—total, unruptured, and ruptured subgroups), in terms of Op-Mo, PND, La-Mo, and Re-int. Moreover, new studies, focused on different subgroups (saccular and non-saccular aneurysms) and different techniques, might be of benefit.

**Table 4:** Conclusion on the comparison between coiling and clipping, on the management of posterior circulation IAs (total, unruptured, and ruptured subgroups), in terms of Op-Mo, PND, La-Mo, and Re-int

Group population	Op-Mo	PND	La-Mo	Re-int
Total aneurysms	Not statistical significant	Coiling	Not statistical significant	Clipping
Unruptured aneurysms	Lack of data	Not statistical significant	Lack of data	Lack of data
Ruptured aneurysms	No statistical significant	Not statistical significant	Lack of data	Lack of data
Conclusion	Case by case management	Coiling	Case by case management	Clipping

## Abbreviations

AICA: Anterior inferior cerebellar artery
BA: Basilar artery
CI: Interval of confidence
IAs: Intracranial aneurysms
ISAT: International Subarachnoid Aneurysm Trial
ISUIA: International Study of Unruptured Intracranial Aneurysms
La-Mo: Late mortality
NOS: Newcastle Ottawa Scale
Op-Mo: Operative mortality
OR: Odds ratio
PCA: Posterior cerebral artery
PICA: Posterior inferior cerebellar artery
PND: Permanent neurologic deficit
PRISMA: Preferred Reporting Items for Systematic Reviews and Meta-Analyses
Re-int: Re-intervention
SAH: Subarachnoid hemorrhages
SCA: Superior cerebellar artery
VA: Vertebral artery
