# Publisher Correction to: Chinese Neurosurgical Journal, volume 5

**DOI:** 10.1186/s41016-019-0169-4

**Published:** 2019-08-08

**Authors:** Chinese Neurosurgical Journal

**Affiliations:** London, UK

**Publisher correction to: Chinese Neurosurgical Journal (2019) 5**

**https://doi.org/10.1186/s41016-019-0163-x**

An error occurred during the publication of one article in *Chinese Neurosurgical Journal*. This article was published in volume 5 with a duplicate citation number.

In this correction article the old and new citation metadata are published in Table 1.

**Table 1:** Overview of incorrect and correct citation metadata

DOI	Incorrect citation number	Correct citation number
10.1186/s41016-019-0163-x	1	16

The original article has been updated. The publisher apologizes for the inconvenience caused to our authors and readers.

